# The role of interpersonal coordination dynamics in alliance rupture and repair processes in psychotherapy—A systematic review

**DOI:** 10.3389/fpsyg.2023.1291155

**Published:** 2024-01-04

**Authors:** S. S. Høgenhaug, M. T. Kongerslev, G. Kjaersdam Telléus

**Affiliations:** ^1^Clinic North, Psychiatric Hospital, Bronderslev, Denmark; ^2^Department of Clinical Medicine, Faculty of Medicine, Aalborg University, Aalborg, Denmark; ^3^Department of Psychology, University of Southern Denmark, Odense, Denmark; ^4^Mental Health Services West, Region Zealand, Slagelse, Denmark; ^5^Psychiatry, Aalborg University Hospital, Aalborg, Denmark; ^6^Psychology, Department of Communication and Psychology, Aalborg University, Aalborg, Denmark

**Keywords:** interpersonal coordination, process research, psychotherapy, therapeutic relationship, rupture, repair

## Abstract

**Introduction:**

The purpose of this systematic review is to expand our knowledge of the underlying mechanisms of the alliance in psychotherapy. This is done by examining the association between alliance rupture and repair processes and interpersonal coordination dynamics.

**Method:**

A systematic review based on PRISMA guidelines was conducted, aimed at papers investigating the association between alliance rupture and repair episodes and different behavioral modalities (i.e., physiology, movement) in the psychotherapeutic interaction. Seventeen studies were included for full text-analysis.

**Results:**

The results indicate that rupture and repair episodes were associated with interpersonal coordination dynamics. Different modalities (movement, heart rate, and vocalization) were found to serve as markers for alliance rupture and repair events. Facial expressions, physiological arousal, vocalization, and behavior were found to play important roles in the therapeutic interaction in relation to mutual emotion regulation, empathic response, safety, trust, and meaning-making.

**Discussion:**

Limitations of this review are discussed, including the great methodological variation and selection bias observed in the reviewed studies. Recommendations for future research in this area are presented. Overall, interpersonal coordination dynamics was found to have the potential to help identify and manage alliance ruptures and foster repairs in the therapeutic interaction, which has high potential for future clinical work and training.

## 1 Introduction

The working alliance has relatively consistently been found to be a robust predictor for outcome across a wide range of different psychotherapies and mental health problems (Del Re et al., 2012; Wampold, 2015; Flückiger et al., 2018; Bo et al., 2022). In a meta-analysis that included 295 studies, with data on more than 30,000 patients, a stronger alliance was found to be associated with better treatment outcome (*r* = 0.28, corresponding to a medium effect size of Cohen’s d = 0.58) (Flückiger et al., 2018). Also, studies have found that a weak alliance is associated with poorer outcome and unilateral termination on behalf of the patients (Martin et al., 2000; Horvath, 2006). Referred to variously as the working alliance (Bordin, 1979; Greenson, 2008), helping alliance (Alexander and Luborsky, 1986), real relationship (Gelso, 2009), and therapeutic alliance (Rogers, 2012), among others, research no doubt indicate that the alliance is closely associated with positive treatment outcome in psychotherapy, supporting the persistent examination of alliance processes in psychotherapy research (Karver et al., 2018; Flückiger et al., 2020). The first decade of alliance research primarily focused on the association between early session ratings of the alliance and treatment outcome, operationalized as pre-post-treatment symptom reduction. Studies consistently found an association between a strong early alliance and treatment outcome (Horvath and Symonds, 1991). Nevertheless, showing that psychotherapy is effective and that the alliance is a strong predictor of outcome does not provide evidence about why and how the treatment works (Kazdin, 2007). In the attempt to elucidate change mechanisms in psychotherapy, the past two decades of alliance research has tried to clarify the underlying mechanisms of the alliance. Great effort has been made to uncover interactional patterns, specific ingredients, and process markers in the therapeutic interaction (Antichi and Giannini, 2023), both within sessions (Ramseyer, 2020) and over the course of treatment (Luo et al., 2022). This knowledge is particularly important to gain more in-depth insight into what lead to change and why. Moreover, increased understanding of alliance processes is crucial in critical situations during the therapeutic interaction when the therapeutic work malfunctions as this could help guide interventions and therapeutic progress, and potentially prevent harmful effect and premature drop out (Cuijpers et al., 2019). Despite decades of research attempting to understand the complexity of the psychotherapeutic alliance (Norcross and Wampold, 2011), the processes that influence the quality of the alliance are still not well understood. Thus, knowledge in this area is inconclusive and calls for further attention to enrich our insight into what drives psychotherapeutic change (Koole and Tschacher, 2016; Kleinbub et al., 2020).

As the concept of the alliance has both been defined and operationalized differently throughout history, what is meant by the alliance when used in this paper is initially clarified. This review draws on the central alliance work of Safran and Muran (2000b). Safran and Muran (2000a) conceptualize the alliance as a dynamic process of intersubjective negotiation where both the patient and the therapist contribute to the collaboration in the therapeutic process. Inspired by Bordin (1979) transtheoretical reformulation of the alliance construct, the alliance is broadly defined as mutual agreement on goals, assignment of tasks, and the development of an emotional bond between patient and therapist (Bordin, 1979). Deterioration in any of these dimensions are described to create disharmony in the collaboration and might cause critical disruptions in the therapeutic process. In their elaboration of the alliance, Safran and Muran (2000a) have put special attention to cycles of ruptures and repairs during the therapeutic interaction. Ruptures occur when there is disagreement on goals of treatment, inability to work collaboratively on the tasks of treatment, or if there are strains in the relational bond (Eubanks et al., 2018). Ruptures are classified in two ways: 1) Confrontational ruptures, where the patient or therapist moves against the other, shows dissatisfaction, or disagreement, and 2) withdrawal ruptures, where the patient or therapist moves away from the other and disengages from the therapeutic process (Eubanks et al., 2015). The alliance is believed to fluctuate in sequences of ruptures and repairs over the course of treatment rather than being stable once it has been established. The nature of rupture representations has been shown to vary considerably in intensity and number over the course of treatment (Safran and Muran, 2006). They can manifest as either minor tensions or full blown breakdowns in the continuous conscious and unconscious negotiation of the patient’s and therapist’s needs, desires, and intentions in the interaction (Eubanks et al., 2015). According to Eubanks-Carter et al. (2014), a rupture can be defined as repaired when the emotional bond is restored and the patient and therapist have resumed collaboration on tasks and goals. Rupture and repair processes referring to the re-establishment of the alliance is considered an important change mechanism (Eubanks et al., 2021).

Ruptures are believed to be inevitable in treatment and can be considered as stressful events challenging the alliance and progress of treatment. Working through alliance ruptures involves oscillations between affective misattunement and attunement (Safran et al., 2011). They have been described as transference-countertransference enactments, resulting from miscoordination between self and mutual-regulation in the interaction (Beebe, 2006). During the therapeutic interaction, the patient, but also the therapist, tend to repeat dysfunctional interpersonal behaviors, often creating in-session moments of tension (Safran and Kraus, 2014). Mutual efforts to recognize and address ruptures are shown to lead to collaborative exploration on these moments within the sessions elaborating on the patient’s underlying intentions, feelings, and needs which are shown to foster growth and insight (Safran and Muran, 2000b). Studies have found resolution to be associated with corrective experiences (Eubanks et al., 2021), therapeutic change (Zilcha-Mano and Errázuriz, 2017), and better outcome (Eubanks et al., 2018). Poor handling of rupture events is related to premature drop out (Tryon and Kane, 1995; Coutinho et al., 2014a) and unresolved difficulties or deterioration (Castonguay et al., 2010). The link between rupture-repairs and outcome has continuously been demonstrated across different therapies, diagnoses, and therapists, indicating that rupture and repair processes are a common factor important for psychotherapeutic change (Stiles et al., 2004; Strauss et al., 2006; Cash et al., 2014; Boritz et al., 2018; Gersh et al., 2018). Additionally, rupture repair processes are trans-theoretical, and knowledge about the dynamic complexity and management of such events are relevant to clinicians, no matter their psychotherapeutic orientations (Safran and Kraus, 2014). This highlights the importance of examining rupture repair processes when seeking to uncover the processes of alliance development and improve effectiveness. Even though several studies have emerged showing the association between alliance ruptures, repairs, and outcome, research is sparse in relation to the underlying processes by which ruptures and repairs occur (Altimir and Valdés-Sánchez, 2020; Mylona and Avdi, 2021). Thus, the therapeutic value of gaining more knowledge of moment-to-moment alliance fluctuations and expanding insight into critical moments during therapeutic interactions may offer valuable insight into interactional processes involved in driving therapeutic change which could be integrated into clinical practice in the future.

However, examining rupture repair processes is complex, and different approaches have been widely discussed within research. Thus, alliance rupture repair processes have been assessed using multiple research methods including questionnaires from the patient and/or therapist to assess shifts in alliance quality within sessions, questionnaires measuring the alliance quality over the course of treatment (Muran et al., 2009), and observer-based ratings of transcriptions and videos of therapy sessions (Eubanks et al., 2015). In the assessment of ruptures and repair, disagreements about the identification of rupture repair processes have been discussed in relation to defining the concepts (Safran and Muran, 2000b), and how to determine how much intensity a rupture should include to be considered a rupture (Safran and Muran, 2006). Methods highly rely upon subjective and explicit observations of the therapist, patient, and/or observer. Argumentation has been made that current assessment methods might have a tendency to emphasize the explicit collaboration, and deemphasize the focal role of implicit and unconscious factors in the therapeutic interaction (Safran and Muran, 2006). This leaves space for further methodological development, especially regarding interactional processes hard to identify from at subjective perspective (Friedman, 2020).

In this review, Interpersonal Coordination Dynamics (ICD) is examined as a possible way to shed new light on the more implicit alliance processes. However, studying ICD is complex, and the terminology surrounding it is fragmented. Several operationalized forms of the concept have been proposed, including synchronization (Paulick et al., 2018a), attunement (Rocco et al., 2017), and interpersonal physiology (Kleinbub, 2017). As such, a broad definition is chosen to limit the possibility of excluding relevant studies in the search process (Kelso, 2009). Overall, ICD can be defined as the temporal coordination of interacting partner’s behavioral and physiological function in the here-and-now of a communication context together with verbal content (Koole et al., 2020). ICD is a natural interpersonal phenomenon of how people’s behavioral, physiological, and affective experiences and reactions tend to spontaneously appear simultaneously (Koole and Tschacher, 2016). We might also add that such spontaneously interpersonal coordination appears to be normal and important for interpersonal encounters from a developmental perspective (Beebe and Lachmann, 2020). ICD differs from mimicry or imitation, as it depends on the mutual timing of the therapist’s or patient’s response, no matter the precise form of these responses. For example, the therapist can show a hand gesture in response to the patient’s shaking his head, which also classifies as synchronization (Koole and Tschacher, 2016). Thus, ICD refers to the degree to which behavioral and physiological responses in the therapeutic interaction are patterned, non-random, and/or synchronized in relation to timing and form (Kelso, 2009). Whenever people interact, they tend to spontaneously synchronize their behavioral responses (Repp and Su, 2013). This process of behavioral coordination between them inclines an adaptation to each other’s rhythms and cycles of behavior, which has been found to be crucial for interpersonal functioning due to higher corporation, rapport, and adaptive emotion regulation in different types of relationships (Wiltermuth and Heath, 2009; Vacharkulksemsuk and Fredrickson, 2012; Aafjes-van Doorn and Müller-Frommeyer, 2020). The emergence of the interpersonal coordination paradigm in psychotherapy research is an innovative line of inquiry, as it has the potential to broaden our understanding of meaningful implicit, non-conscious, dyadic processes such as the therapeutic alliance, attunement, empathy, interpersonal coordination, synchrony, and mutual regulation (Atzil-Slonim and Tschacher, 2020; Kleinbub et al., 2020). Based on the theoretical framework of embodied cognition and embodiment in psychotherapy, which stresses the significance of considering mental processes as grounded in the body, and situated in the environment (Trasmundi et al., 2023), ICD research emphasizes behavioral modalities. In doing so, ICD research expands our understanding of interactional patterns in the alliance beyond verbal aspects (Semin and Cacioppo, 2008; Ramseyer, 2011; Koole et al., 2020; Mende and Schmidt, 2021).

In psychotherapy research, ICD is considered a fundamental mechanism underlying the therapeutic alliance. For instance, Wiltshire et al. (2020) found ICD to be associated with outcome and different important areas of functioning, such as empathic response (Marci and Orr, 2006), the real relationship (Peluso et al., 2012), and providing feelings of safety that are conductive for progress and learning (Geller, 2019). Furthermore, various modalities (Burgoon et al., 2002), have been suggested as potentially important markers of the alliance including physiological synchrony (Kleinbub et al., 2020), vocal synchrony (Schoenherr et al., 2021), linguistic markers (Goldberg et al., 2020), biological markers (Zilcha-Mano et al., 2020), and movement markers (Ramseyer and Tschacher, 2016; Paulick et al., 2018a). To specify, modalities refer to categories of expressive behavior and are channels or modes of behavioral communication, including voice, facial expressions, movement, physiology etc. Dyadic synchrony between patient and therapist is usually assessed by calculating the association or interdependence between the interacting partner’s signals in these different modalities over time (Atzil-Slonim et al., 2023). While positive associations between ICD and the therapeutic alliance have been repeatedly identified (Ramseyer and Tschacher, 2011; Altmann et al., 2020), the field is still in its infancy and is showing inconsistency. Hence, some studies have not found associations between ICD and patient/therapist-rated alliance (Paulick et al., 2018a; Ramseyer, 2020). The role and functionalities of ICD during the therapeutic interaction is still limited and calls for further investigation. Nevertheless, an advantage of applying ICD to the research on rupture and repair processes is that it provides a common language for investigating several kinds of behaviors in both the therapist and patient, divergent from investigating specific behaviors in rupture repair episode unique for the patient or the therapist. Thus, this might allow bridging rupture and repair episodes to crucial interpersonal constructs and functionalities (Luo et al., 2021).

Following the growing body of research examining interactional processes involved in building, negotiating, and repairing the alliance (Eubanks et al., 2018; Kleinbub et al., 2020), the aim of this review is to synthesize current findings from studies examining the link between rupture and repair processes and ICD in the therapeutic interaction to further our understanding of the dynamic nature of the alliance (Wampold, 2015; Hofmann and Hayes, 2019; Norcross and Lambert, 2019; Friedman, 2020). In sum, there are several reasons why these two areas are considerably relevant to combine. As pointed out, combining the fields allow to go beyond the tendency of prior research to primarily focus on the subjective aspects of the alliance, as explicated in the words or experiences of patient and therapist. Thus, allowing an investigation of more implicit—and for the most part unconscious or automatic—interactional processes of the patient’s and therapist’s behavioral and physiological co-regulation (Fonagy and Allison, 2014; Koole and Tschacher, 2016; Bo et al., 2017). Both research fields acknowledge that the alliance is built, negotiated, and repaired by the mutual but asymmetrical interaction between patient and therapist. Both are reciprocally influencing and affecting each other and enabling the examination of how both are transformed and shaped over time in the therapeutic process (Koole and Tschacher, 2016; Muran et al., 2019; Atzil-Slonim and Tschacher, 2020). Whilst research combining the two areas are still in its early phase, such research could enable the development of key knowledge and valuable tools for detecting important moment-to-moment regulatory processes occurring between patient and therapist (Kleinbub et al., 2020). If successful, this could have a significant impact on the clinical process and could offer new, time-saving, automatic methodological approaches relevant for both clinical practice and research.

## 2 Materials and methods

This systematic review was conducted based on the principles of the Preferred Reporting Items for Systematic Reviews and Meta-Analyses (PRISMA) statement (Page et al., 2021). The study was registered to the International Prospective Register of Systematic Reviews (PROSPERO) prior to onset. Code: CRD42023375031.

### 2.1 Literature search

Relevant studies were identified in a literature search on research published prior to June 2023 using Psychinfo, Embase, Scopus, and PubMed online databases. The search terms were based on previous studies, and literature reviews from both the field of ICD and alliance rupture and repair processes (Koole and Tschacher, 2016; Eubanks et al., 2018; Wiltshire et al., 2020). Additionally, it was informed by a preliminary search of the literature in an early stage of the review, prior to conducting the final database search.

Four sets of keywords were chosen to identity the pertinent papers: relationship (relation* OR alliance* OR interaction* OR interpersonal*), rupture repair (rupture* OR repair* OR tension* OR resolution* OR resolve* OR confrontat* OR negotiat* OR conflict* OR deterioration*), therapist (therapist* OR psychotherapist* OR psychologist* OR psychiatrist* OR analyst), coordination (synchron* OR coordinat* OR covariation* OR coheren* OR linkage* OR contagion* OR attune* OR align* OR concordance* OR mirror* OR “language style” OR facial OR “skin conductance” OR “skin temperature*” OR respirati* OR “heart rate” OR “speech rate” OR vocal OR pitch OR non-verbal OR “non-verbal” OR “body movement” OR “body language*” OR eda OR vocalizat* OR gestur* OR acoustic* OR dermal* OR “autonomic nervous system” OR “physiological arousal” OR biomarker*) (see Supplementary Appendix 1 for full search strategy in each database).

The first author (SSH), a chief librarian, along with a second librarian, at Aalborg Psychiatric Hospital Library performed the search. The search strategy was applied to abstract, title, and medical subject headings (MESH terms) in each database. It was adapted and modified according to the specific advanced search features of each database. A reversed citation (i.e., cited by) was used in Google Scholar and Scopus for all included articles to identify potential articles not captured by the databases. An additional search in the databases was performed just before submission to ensure newly published work was captured. The first search was performed in December 2022 and the second search was performed in June 2023. The searches returned 2,289 articles in total and 1,262 unique titles and abstracts for screening, after removing duplicates (*n* = 1,027). Endnote (Bramer et al., 2017) was used as a management tool to remove duplicates before screening. The online review program Ryyan (Ouzzani et al., 2016) was used as a technical tool to blind screening and rating of the studies for inclusion and exclusion.

### 2.2 Study selection

As the field of interest is rather new, a broad inclusion approach was applied to achieve the aim of this review. Overall, this review includes any direct or indirect assessment tools of alliance rupture and repair processes and any modalities of interpersonal coordination in the psychotherapeutic interaction (i.e., movement, vocalization, physiology). Additionally, as the association between rupture repairs and ICD is the focus of attention, a broad population of individuals suffering from various major or minor difficulties are included as well as any psychotherapeutic method and therapy modality. This integrative approach has the potential to consolidate, nuance, and advance our understanding of the associations between ICD and alliance rupture and repair processes. Studies included in this review were selected based on the following more specific inclusion and exclusion criteria:

Inclusion criteria was empirical examination of dyadic psychotherapy sessions between adult patients and therapists (or pseudo-interactions, defined as interactions based on realistic patient-therapist interactions); must include quantitatively assessed vocal, hormonal, behavioral, and/or physiological measurements recorded at least two times for each therapy session; must include direct or indirect measurements of alliance rupture and repair processes, and must be written in English and published in international peer-reviewed journals.

Exclusion criteria was studies of psychotherapy patients if there are no analyses derived from actual sessions or interviews (e.g., if the analyses were conducted on patients performing laboratory-style tasks); studies of couples and group therapy; studies of children; studies of clients not suffering from mental distress or personal issues; studies examining only interpersonal coordination dynamics on questionnaires; studies not aimed at studying alliance rupture and repair processes.

**FIGURE 1:**
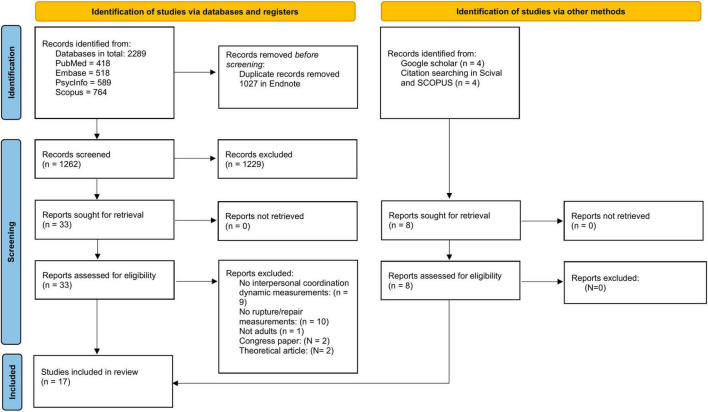
PRISMA flow diagram for study selection (Page et al., 2021).

Prior to commencing the blinded screening process, a random sample of 20 abstracts from a try search was screened by the first author (SSH) and a research assistant to ensure reliability in correctly selecting articles for inclusion. Afterward, the final screening was conducted on titles and abstracts. Final eligibility of the studies was assessed on full text screening based on the above written inclusion and exclusion criteria. The first author and a research assistant screened the studies separately and synthesized their findings. If disagreement, the studies were discussed based on inclusion and exclusion criteria to reach agreement. When in doubt, a third reviewer (the last author) was consulted. The initial screening resulted in a total of 33 studies from the database search, four studies from the citation search, and four studies from a free Google Scholar search. Full text screening was performed on 41 studies. Eleven studies were included from the Ryyan screening and eight studies were included from the other sources. Primary reasons for exclusion were missing measurements of ICD (n = 9) or alliance rupture and repair processes (*n* = 10). Also, two studies were excluded because they were theoretical papers and two papers were excluded, as they were congress papers and not peer-reviewed published studies. Seventeen studies were found suitable for inclusion (see Figure 1 for selection process).

### 2.3 Quality of studies

The quality of the included studies was verified in accordance with The Joanna Briggs Institute (JBI) Critical Appraisal Checklist. This tool presents questions evaluating different quality items in the studies, which should be answered with a “Yes”, “No”, “Unclear”, or “Not Applicable”. Each “yes” answer corresponds to one point and each “no”, “unclear” and “not applicable” corresponds to 0 points. The JBI does not differentiate between studies of low and high quality but provides a total score for each article based on the number of “Yes”. As the included papers primarily consisted of case reports and case series, two questionnaires from the JBI were chosen for quality assessment; 1) The Critical Appraisal Checklist for case reports and 2) The Critical Appraisal Checklist for case studies (Munn et al., 2020). The JBI checklist for case reports is rated on a scale ranging from 0 to 8, and the checklist for case studies ranges from 0 to 10. Each included study was rated for each question and given 1 point if the study fulfilled a question and 0 points if the question was rated no, unclear, or not applicable (Munn et al., 2014; DeLang et al., 2019). Two raters, the first author (SSH) and a research assistant, rated each study independently followed by a synthesizing of their findings. If disagreement, results were discussed with a third reviewer (GKT) to reach the final response. A calculation of weighted kappa showed good interrater agreement (case series: κ = 0.85, case reports: κ = 0.94) (Cohen, 1968). Because of the limited findings in this area so far, all matching articles were retained no matter their methodological quality or year of publication. An overview of risk of bias is included in Table 1.

**TABLE 1:** Key findings.

References	Country	Patient	Mental distress/ issues	Therapist	Session data, therapy type, and setting	Modality	ICD method	Rutpure/ resolution measure	Quantification of Rupture/resolution and ICD	Primary results	Risk of bias
Mylona and Avdi (2021) *	Greece	1	moderate to severe distress	1	1 session psychoanalytic therapy naturalistic setting	Absolute Stress Vector (ASV)	Partial Directed Coherence	Rupture resolution System (3RS)	Dividing into 50-s time windows, and using a non-linear lagged regression analysis	Synchronization helped locate in-session events with ruptures and repairs. Increased physiological arousal was found to accompany a shift from disruption to negotiation within session.	8
Luo et al. (2022)	USA	8	personality disorder (*N* = 5), major depressive disorder (3)	8	16 sessions interpersonal psychodynamic psychotherapy naturalistic setting	Dominance and Warmth	Continuous Assessment of Interpersonal Dynamics (CAID). Complementarity was calculated as the cross-correlation between two time-residualized time series of therapist’s and patient’s warmth or dominance for each 30-s segment	Rupture resolution System (3RS)	Cross-correlation between two time-residualized time series of Therapist and Patient warmth and dominance for each 30 sec., Group Iterative Multiple Model Estimation (GIMME), and Unified Structural Equation Modeling (uSEM)	Heterogeneity was found in the association between interpersonal behaviors and ruptures. Subgroup analysis of 7 sessions revealed that therapist’s dominance negatively predicted concurrent withdrawal ruptures. Subgroup analysis of 2 sesions from the same dyad identified how confrontation ruptures predicted concurrent dominance complementarity positively.	7
Deres-Cohen et al. (2021)	Israel	75	depression	8	418 sessions psychodynamic psychotherapy naturalistic setting	Behavior (non-verbal synchrony)	Motion energy analysis (MEA)	Rupture resolution System (3RS)	Time-lagged cross-correlation + −5s., and Multilevel model	Confrontation ruptures were found to be significantly associated with non-verbal synchrony within sessions.	7
Mylona and Avdi (2021) *	Greece	7	depression, anxiety and interpersonal difficulties	2	12 sessions psychodynamic psychotherapy naturalistic setting	Absolute Stress Vector (ASV)	Separate analysis were conducted for patient’s and therapist’s ASV using R version 3.6.1 and Ime function from the Nlme package: Non-Linear Mixed effects.	Rupture resolution System (3RS)	Multilevel model	No significant differences were identified between client’s arousal in rupture and no rupture episodes. Mixed ruptures were associated with client’s higher physiological arousal compared to no rupture episodes and compared to both confrontation and withdrawal ruptures. Segments with confrontational ruptures were associated with client’s lower arousal compared to no rupture segments.	5
Luo et al. (2021)	USA	1	interpersonal difficulties	3	3 sessions client-centered therapy, gestalt therapy rational-emotive behavior therapy demonstration videos	Dominance and Warmth	Continuous Assessment of Interpersonal Dynamics (CAID)	Rupture resolution System (3RS)	Dynamic Structural Equation Modeling (DSEM)	The study found dyadic-specific interpersonal patterns of associations between warmth and dominance and rupture segments.	4
Christian et al. (2021)	USA	27	personality disorder, cluster C	27	27 sessions brief relational psychotherapy naturalistic setting	Linguistics	Weighted Referential Activity Dictionary (WRAD)	Segmented Working Alliance Inventory (SWAIO)	Pearson correlation analysis	During ruptures decrease in emotional engagement and increase in intellectualization was observed for the patient and the therapist when compared to no-rupture events.	7
Aafjes-van Doorn and Müller-Frommeyer (2020) **	USA	1	narcissistic	1	7 sessions rogerian psychodynamic psychotherapy pseudo-interaction	Language Style Matching (LSM)	Language Style Matching (LSM), Linguistic Inquiry and Word Count, reciprocal LSM	Rupture resolution System (3RS), Segmented Working Alliance Inventory (SWAIO)	Multilevel model, and linear mixed effect model	LSM was found to positively predict the frequency of ruptures.	5
Del Giacco et al. (2020)	Spain	7	depressed symptomato-logy	1	20 sessions psychodynamic psychotherapy naturalistic setting	Verbal, vocal, interruption modes	The Communicative Modes Analysis System in Psychotherapy (CMASP)	Collaborative Interaction Scale-Revised (CIS-R)	Lag sequential analysis, and Polar coordinate analysis	Specific verbal and non-verbal modes for the patient and for the therapist were found to be significantly connected to the reciprocal construction of the alliance.	8
Zilcha-Mano et al. (2020)	Israel	1	major depressive disorder, interpersonal difficulties	1	4 sessions supportive expressive psychotherapy naturalistic setting	Oxytocin (OT)	OT-Enzyme-linked immunosorbent assay (ELISA)	Rupture resolution System (3RS)	Pearson correlation analysis	Evidence of ruptures were noticable in alliance rupture and repair ratings, questionaire data (WAI), and oxitocin measures. Oxytocin was found to be able to serve as a biomarker for the therapeutic alliance.	5
Altimir and Valdés-Sánchez (2020)	Chile	1	depressive symptoms and interpersonal difficulties	1	30 session short term psychodynamic psychotherapy naturalistic setting	Facial expression, Verbal Relational Offers	Facial Action Coding System (FACS) and Qualitative Content Analysis	Rupture resolution System (3RS)	Hierarchical Linear Modeling (HLM)	The study found characteristic patient-therapist facial-verbal patterns related to rupture and resolution events.	5
Avdi and Seikkula (2019) *	Greece	1	moderate to severe distress	1	1 session psychoanalytic therapy naturalistic setting	Absolute Stress Vector (ASV)	Unknown	Rupture resolution System (3RS)	Unknown	Embodied attunement was found to be highest during rupture segments. The therapeutic dyad was found to be in sync until tension was resolved.	8
Zalman et al. (2019) **	USA	1	narcissistic	1	7 sessions rogerian psychodynamic psychotherapy Pseudo-interaction	Language Style Matching (LSM)	Language Style Matching (LSM), Linguistic Inquiry and Word Count	Rupture resolution System (3RS), Working-Alliance Inventory Observer scale (WAI-O)	Calculating total Language Style Matching (LSM Total) = (LSM word category 1 + LSM word category 2 + …. + LSM word category 9)	Unconscious aspects of the alliance (measured with LSM) was found to start deteriorate just before ruptures occured.	6
Negri et al. (2019)	Italy	40	panic disorder (*N* = 4), agoraphobia (*N* = 3), specific phobia (*N* = 1), obsessive compulsive disorder (*N* = 6), obsessive compulsive personality disorder (*N* = 2), bulimia nervosa (*N* = 5), anorexia (*N* = 2), binge eating disorder (*N* = 1), major depressive disorder (*N* = 8), non-clinical group (*N* = 8)	1	40 sessions unknown naturalistic setting	Linguistics	Weighted Referential Activity Dictionary (WRAD)	Segmented Working Alliance Inventory (SWAIO)	Pearson correlation analysis	During ruptures compared to no-rupture segments, the therapist and patient had speech marked by fewer disfluencies. A stronger alliance at the end of the session was predicted by speech in the middle of the session for the patient.	7
Rocco et al. (2017)	Italy	2	panic disorder and interpersonal difficulties (*N* = 1), non-organic sexual problems and interpersonal difficulties (*N* = 1)	1	2 sessions short term psychodynamic psychotherapy naturalistic setting	Speech rate	Verbal Attunement Index (VAI)	Collaborative Interaction Scale (CIS)	Speech rate (SR) (VAI = SRp/SRt): When VAI is closer to 1 non-verbal attunement is higher. Rupture/resolution: higher session quality index (SQI) indicates lower level of collaboration and lower SQI indicates higher level of collaboration	A good outcome session was found to be associated with significant higher levels of attunement in ruptures and resolutions when compared to a poor outcome session.	6
Morán et al. (2016) ***	Chile	5	adaptive disorder (*N* = 2), anxiety disorder (*N* = 1), depression (*N* = 1), personality disorder (*N* = 1)	5	67 change episodes, 86 rupture episodes psychodynamic psychotherapy cognitive behavioral psychotherapy naturalistc setting	Vocal Quality Pattern, Facial Expression and Discursive Position	Vocal Quality Pattern (VQP), Facial Action Coding System (FACS) and discursive position (DP)	Rupture resolution System (3RS)	Logistic Hierachical Regression Analysis	The study found correspondence between rupture events and the use of characteristic vocal quality patterns and facial expressions which was related to specific regulatory strategies within sessions.	4
Tomicic et al. (2015) ***	Chile	5	adaptive disorder (*N* = 2), anxiety disorder (*N* = 1), depression (*N* = 1), personality disorder (*N* = 1)	5	67 change episodes, 86 rupture episodes psychodynamic psychotherapy cognitive behavioral psychotherapy naturalistic setting	Vocal Quality Pattern (VQP)	Vocal Quality Pattern (VQP)	Rupture resolution System (3RS)	Logistic Hierachical Regression Analysis	8 recurrent and stable discourse-voice regulatory strategies of the patients and three of the therapists were identified during rupture episodes.	4
DiMascio et al. (1957)	Unknown	1	unknown	1	12 Sessions unknown naturalistic setting	Heart rate, skin conductance	Correlation analysis	Bales’ method for disagreement	Rank order correlation	Higher patient rated tension scores was found to correlate with higher heart rate for both the patient and therapist. The patient showed tension more through heart rate than through skin conductance.	2

*These studies are based on the same population.

** These studies are based on the same population.

*** These studies are based on the same population.

### 2.4 Data extraction

Data was extracted on the following information from each of the studies: (1) reference, (2) country, (3) patient (N), (4) mental distress/issues, (5) therapist (N) (6) session data, therapy type, and therapy setting, (7) modality, (8) ICD assessment method, (9) rupture resolution assessment method, (10) quantification of rupture resolution and ICD, and (11) key findings. Excel was used for data extraction. Initially, the first author (SSH) conducted the data extraction. A research assistant checked the results to minimize errors.

### 2.5 Data synthesis

Results from the included studies were synthesized and summarized narratively. Initially, a meta-analysis was planned if possible, but because of the heterogeneity of the included studies (sample sizes, modalities, measurement methods, experimental design), it was not found meaningful to perform (Page et al., 2021).

## 3 Results

Initially, the results from this systematic review will be presented as a summarizing narrative of study characteristics to provide solid contextualization of the included studies. Secondly, as the findings revealed significant methodological heterogeneity, a methodology run-through is offered followed by a section on study quality ratings to provide an informal foundation before finally evaluating the results regarding the association between rupture repair and ICD. A synthesized overview of the findings is presented in Tables 1, 2.

**TABLE 2:** Functionalities.

References	Modality	Regulatory characteristics	Functionality
Mylona et al. (2022)	Physiological arousal (ASV)	Management of rupture episodes where characterized by the therapist’s use of metacommunication and validation combined with smile, gaze, gentle tone, and nods.	Emotion regulation, affiliation, empathy, meaning making
Avdi and Seikkula (2019)	Physiological arousal (ASV)	Attunement in physiological arousal and postural congruence during ruptures where found to foster new corrective experiences and facilitate relational spaces of “being” with another person.	Emotion regulation, facilitates exploration, meaning making, and trust
Mylona and Avdi (2021)	Physiological arousal (ASV)	Patient expressed disagreement about the therapist and therapist’s use of metacommunication were identified as potential promoters of tension release and facilitators of resolution.	Emotion regulation
Morán et al. (2016)	Facial expression, language	Rupture episodes were characterized by contradictory vocal regulatory strategies of the patient including both distancing behavior and emotional engaging combined with decreased eye contact, while the therapist was found to use regulatory strategies through a disaffected position with increased use of adaptors with the purpose of addressing the effect of the ruptures in the sessions	Emotion regulation and meaning making
Altimir and Valdés-Sánchez (2020)	Facial expression	During rupture episodes the therapist was found to use questioning combined with facial-affective behavior of self-soothing, control, and gazing towards the patient. During resolution the patient uses gazing at the therapist combined with self-soothing behavior to regulate inner distress	Emotion regulation
Luo et al. (2022)	Warmth and dominance	Therapist’s being less dominant than usual predicted more concurrent withdrawals in a subgroup. Idiographic results indicated high heterogeneity in the association between ICD and alliance ruptures.	Emotion regulation
Luo et al. (2021)	Warmth and dominance	During rupture episodes several session specific patterns of regulatory strategies where identified between patient and therapist.	Emotion regulation
Negri et al. (2019)	Linguistics	In the initial phase of an intake session it was found that if patient’s made fewer references to body sensory processes, and bodily activities, the alliance was stronger. In the middle phase of an intake session it was found, that patient’s expressing and speaking about their life without intense work of reflection correlated with a strong alliance. In the last phase of an intake session it was found that patient’s longer speech containing less neutral affect words was associated with a stronger alliance	Warmth, safety, and analytic trust fostering elaboration of the patient’s inner experiences
Zalman et al. (2019)	Language style matching	Therapist’s and patient’s were found to expose vulnerability after a period of increased ruptures.	Emotion regulation
Rocco et al. (2017)	Speech rate	ICD was found to be a moderating factor during rupture episodes.	Clinical attunement enforces the alliance and promotes integration of formal thinking processes influencing cognitive and emotional domains
Tomicic et al. (2015)	Language	8 recurrent and stable discourse-voice regulatory strategies of the patient and three of the therapist were identified during rupture episodes.	Generates relational offers that allow emotion regulation
Christian et al. (2021)	Linguistics	Rupture episodes were characterized by therapist’s decreased emotional engagement and increased distancing combined with linguistic patterns of making sense of, and self-disclose their inner experiences	Emotion regulation, trust
Del Giacco et al. (2020)	Verbal, vocal, interruption modes	Therapist’s verbal asking and exploring combined with non-verbal elaboration and cooperatively interruption, and the patient’s verbal asserting and exploring and non-verbal expressing emotions and cooperatively interrupting were found to foster alliance formation	Emotion regulation, alliance construction

### 3.1 Study characteristics

The 17 included studies were based on data from 13 independent samples. Thus, three studies used data from the same research project to examine physiological arousal during rupture and repair segments (Avdi and Seikkula, 2019; Mylona and Avdi, 2021; Mylona et al., 2022). Another two studies provided different analysis of the same five patients from a research project to describe different aspects of verbal and non-verbal expressions of mutual regulation between therapist and patient during rupture episodes (Tomicic et al., 2015; Morán et al., 2016). Finally, two studies examined the same pseudo-interactions for the link between language style matching and rupture and repair events (Zalman et al., 2019; Aafjes-van Doorn and Müller-Frommeyer, 2020). Even though including studies from the same samples contains a potential risk of bias, and limits the generalizability of the findings, inclusion of each study was evaluated to be suitable in this summarizing review. This evaluation was based on the fact that each study was found to represent unique and different ways of investigating the association between rupture repairs and ICD in relation to their integration of different modalities, varying assessment tools, diverse methodology, and varied analyses. This approach allows further opportunity to nuance and broaden the perspectives on the associations between ICD and rupture repair processes and how to assess them.

Altogether, 68 different therapists were included in this review. A total of 26 identified themselves as males and 39 identified themselves as females. Three studies did not provide information about therapist identification in relation to gender (DiMascio et al., 1957; Rocco et al., 2017; Zilcha-Mano et al., 2020). The total number of patients included was 184. In the included studies, 109 identified themselves as females, 73 identified themselves as males, and two identified themselves as non-binaries. Thus, the latter is an underrepresented minority group in this current research.

The following clinical conditions were represented (note that some patients have multiple diagnosis, mental distress conditions, or personal issues and are therefore represented more than once): depression (*n* = 89) (Tomicic et al., 2015; Morán et al., 2016; Negri et al., 2019; Zilcha-Mano et al., 2020; Deres-Cohen et al., 2021; Mylona and Avdi, 2021; Luo et al., 2022), personality disorders (*n* = 37) (Tomicic et al., 2015; Morán et al., 2016; Negri et al., 2019; Christian et al., 2021; Luo et al., 2022), anxiety disorders (*n* = 17) (Tomicic et al., 2015; Morán et al., 2016; Rocco et al., 2017; Negri et al., 2019; Mylona and Avdi, 2021), interpersonal difficulties (*n* = 13) (Rocco et al., 2017; Altimir and Valdés-Sánchez, 2020; Zilcha-Mano et al., 2020; Mylona and Avdi, 2021), eating disorders (*n* = 8) (Negri et al., 2019), adaptive disorders (*n* = 4) (Tomicic et al., 2015; Morán et al., 2016), depressed symptomatology (*n* = 2) (Altimir and Valdés-Sánchez, 2020; Del Giacco et al., 2020), moderate distress (*n* = 2) (Avdi and Seikkula, 2019; Mylona et al., 2022), and non-organic sexual problems (n = 1) (Rocco et al., 2017), a non-clinical group (*n* = 8) (Negri et al., 2019). One study did not provide sufficient information about clinical conditions (DiMascio et al., 1957).

The following differentiations of therapy types, as identified by the study authors, were included (again note that some studies examined more than one therapy type, which means the total number is more than the sum of the included studies in this review): psychodynamic psychotherapy (*n* = 5) (Tomicic et al., 2015; Morán et al., 2016; Del Giacco et al., 2020; Deres-Cohen et al., 2021; Mylona and Avdi, 2021), short term psychodynamic psychotherapy (*n* = 2) (Rocco et al., 2017; Altimir and Valdés-Sánchez, 2020), psychoanalytic therapy (*n* = 2) (Avdi and Seikkula, 2019; Mylona et al., 2022), Rogerian psychodynamic psychotherapy (*n* = 2) (Zalman et al., 2019; Aafjes-van Doorn and Müller-Frommeyer, 2020), cognitive behavioral psychotherapy (*n* = 2) (Tomicic et al., 2015; Morán et al., 2016), brief relational psychotherapy (n = 1) (Christian et al., 2021), interpersonal psychodynamic psychotherapy (n = 1) (Luo et al., 2022), client-centered therapy (*n* = 1) (Luo et al., 2021), gestalt therapy (*n* = 1) (Luo et al., 2021), rational-emotive behavior therapy (*n* = 1) (Luo et al., 2021), supportive expressive psychotherapy (*n* = 1) (Zilcha-Mano et al., 2020). Two studies did not provide the necessary information on therapy type (DiMascio et al., 1957; Negri et al., 2019).

Most of the studies examined naturalistic clinical settings (*n* = 14) (DiMascio et al., 1957; Tomicic et al., 2015; Morán et al., 2016; Rocco et al., 2017; Avdi and Seikkula, 2019; Negri et al., 2019; Altimir and Valdés-Sánchez, 2020; Del Giacco et al., 2020; Zilcha-Mano et al., 2020; Christian et al., 2021; Deres-Cohen et al., 2021; Mylona and Avdi, 2021; Luo et al., 2022; Mylona et al., 2022). Two studies examined pseudo-interactions (Zalman et al., 2019; Aafjes-van Doorn and Müller-Frommeyer, 2020), and one study investigated psychotherapeutic demonstration videos (Luo et al., 2021).

In total, data from 792 sessions was included. Six studies examined sessions from the initial phase of treatment (Rocco et al., 2017; Avdi and Seikkula, 2019; Negri et al., 2019; Del Giacco et al., 2020; Christian et al., 2021; Luo et al., 2021), and one study examined sessions from the final phase of treatment (Mylona et al., 2022). Two studies explored sessions from the initial, middle, and final phase of treatment, but data did not reflect the developmental process for each participant over the course of treatment (DiMascio et al., 1957; Mylona and Avdi, 2021). Finally, seven studies investigated sessions from the full course of treatment (Tomicic et al., 2015; Morán et al., 2016; Zalman et al., 2019; Aafjes-van Doorn and Müller-Frommeyer, 2020; Altimir and Valdés-Sánchez, 2020; Zilcha-Mano et al., 2020; Deres-Cohen et al., 2021). One study did not provide sufficient information about placement of sessions (Luo et al., 2022).

The following modalities were included: vocalization (used as a broad term including language, speech rate, and linguistic features) (*n* = 8) (Tomicic et al., 2015; Morán et al., 2016; Rocco et al., 2017; Negri et al., 2019; Zalman et al., 2019; Aafjes-van Doorn and Müller-Frommeyer, 2020; Del Giacco et al., 2020; Christian et al., 2021), physiological arousal (*n* = 5) (DiMascio et al., 1957; Avdi and Seikkula, 2019; Mylona and Avdi, 2021; Mylona et al., 2022), facial-affective behavior (*n* = 3) (Morán et al., 2016; Avdi and Seikkula, 2019; Altimir and Valdés-Sánchez, 2020), dominance and warmth (n = 2) (Luo et al., 2021, 2022), movement (*n* = 2) (Avdi and Seikkula, 2019; Deres-Cohen et al., 2021), and biological markers (*n* = 1) (Zilcha-Mano et al., 2020). About half of the studies examined one modality (n = 9) (Negri et al., 2019; Zalman et al., 2019; Aafjes-van Doorn and Müller-Frommeyer, 2020; Zilcha-Mano et al., 2020; Christian et al., 2021; Deres-Cohen et al., 2021; Luo et al., 2021, 2022; Mylona and Avdi, 2021), while the rest of the studies integrated more than one modality (n = 8) (DiMascio et al., 1957; Tomicic et al., 2015; Morán et al., 2016; Rocco et al., 2017; Avdi and Seikkula, 2019; Altimir and Valdés-Sánchez, 2020; Del Giacco et al., 2020; Mylona et al., 2022).

### 3.2 Study methodology

As already mentioned, the included studies showed high heterogeneity according to methodology. As such, it was found appropriate to specify this heterogeneity to be better able to evaluate and compare study findings. All studies examined ruptures (*N* = 17), while only some studies included repair ratings (*n* = 5) (Rocco et al., 2017; Avdi and Seikkula, 2019; Zalman et al., 2019; Altimir and Valdés-Sánchez, 2020; Mylona et al., 2022). Most studies examined alliance rupture and repair processes using the Alliance Rupture Resolution Rating Manual (*n* = 12) (in the following, abbreviated to 3RS) (Eubanks et al., 2015) (Tomicic et al., 2015; Morán et al., 2016; Avdi and Seikkula, 2019; Zalman et al., 2019; Aafjes-van Doorn and Müller-Frommeyer, 2020; Altimir and Valdés-Sánchez, 2020; Zilcha-Mano et al., 2020; Deres-Cohen et al., 2021; Luo et al., 2021, 2022; Mylona and Avdi, 2021; Mylona et al., 2022). However, heterogeneity was found regarding rating procedures. Most of the studies using the 3RS followed the recommended instructions by Eubanks et al. (2015) of coding rupture and repair segments every 5 min during a session (n = 9) (Avdi and Seikkula, 2019; Zalman et al., 2019; Aafjes-van Doorn and Müller-Frommeyer, 2020; Altimir and Valdés-Sánchez, 2020; Zilcha-Mano et al., 2020; Christian et al., 2021; Deres-Cohen et al., 2021; Mylona and Avdi, 2021; Mylona et al., 2022). Others rated ruptures every 30 s during sessions (n = 2) (Luo et al., 2021, 2022), or rated rupture events by rating rupture duration from first hint of a rupture until first hint of resolution (n = 2) (Tomicic et al., 2015; Morán et al., 2016). Most studies examined the number of segments with ruptures and repairs (n = 10) (Rocco et al., 2017; Avdi and Seikkula, 2019; Negri et al., 2019; Aafjes-van Doorn and Müller-Frommeyer, 2020; Altimir and Valdés-Sánchez, 2020; Del Giacco et al., 2020; Zilcha-Mano et al., 2020; Christian et al., 2021; Deres-Cohen et al., 2021; Mylona and Avdi, 2021), other studies examined the percentage of rupture segments during sessions (n = 3) (Zalman et al., 2019; Luo et al., 2021; Mylona et al., 2022) or investigated the actual number of rupture and repair events in the sessions (n = 2) (Tomicic et al., 2015; Morán et al., 2016). Finally, one study examined the intensity of the rupture and repair segments during sessions (Luo et al., 2022). Additionally, the included studies diverged according to whether they examined rupture/no rupture variables or specific rupture types (confrontational, withdrawal, and/or mixed ruptures). Besides the 3RS, a variety of other measures were applied to assess rupture and repair episodes, including the Segmented Working Alliance Inventory−Observer Form (SWAIO) (Berk, 2013) (n = 3) (Negri et al., 2019; Aafjes-van Doorn and Müller-Frommeyer, 2020; Christian et al., 2021), the Working Alliance Inventory Observation scale (Horvath and Greenberg, 1989) (n = 1) (Zalman et al., 2019), the Collaborative Interaction Scale (CIS) (Colli and Lingiardi, 2009) (n = 1) (Rocco et al., 2017), the Collaborative Interaction Scale Revised (CIS-R) (Colli et al., 2019) (n = 1) (Del Giacco et al., 2020), and Interaction Process Analysis (Bales, 1970) (n = 1) (DiMascio et al., 1957).

Like the methodology on 3RS, high heterogeneity was found regarding quantification of ICD. Most commonly, different types of correlation analyses were applied (DiMascio et al., 1957; Zilcha-Mano et al., 2020; Christian et al., 2021; Deres-Cohen et al., 2021; Luo et al., 2021, 2022; Mylona and Avdi, 2021), including traditional correlation measures (DiMascio et al., 1957), Pearson correlation analysis (Zilcha-Mano et al., 2020; Christian et al., 2021), time-lagged cross-correlation analysis, using a mean of all absolute correlation values as the quantity of coordination between patient and therapist (Deres-Cohen et al., 2021), and calculating the complementarity of warmth and dominance between patient and therapist as the cross-correlation between two time-residualized time series for each 30-s segments (Luo et al., 2021, 2022). Ten studies utilized quantitative models to calculate the link between ICD and alliance rupture and repair episodes using multi-level models (n = 3) (Aafjes-van Doorn and Müller-Frommeyer, 2020; Deres-Cohen et al., 2021; Mylona and Avdi, 2021), logistic hierarchical regression analysis (n = 3) (Tomicic et al., 2015; Morán et al., 2016; Altimir and Valdés-Sánchez, 2020), dynamic structural equation modeling (DSEM) (n = 1) (Luo et al., 2021), group iterative multiple model estimation (GIMME) (n = 1) (Luo et al., 2022), polar coordinate analysis (n = 1) (Del Giacco et al., 2020), Pearson correlation analysis (n = 1) (Negri et al., 2019), and rank order correlations (n = 1) (DiMascio et al., 1957).

### 3.3 Study quality

Based on previous recommendations, the Critical Appraisal Checklist for case reports was used when a study included 1–2 cases, and the Critical Appraisal Checklist for case studies was used when a study included 3–10 cases (Nissen and Wynn, 2014). Three studies did not fall into any of the two categories of either case study or case series. One of the included studies was a mixed method study including 75 cases with an in-depth analysis of one case. In this study, it was decided to rate the reported data from the case example as a case report (Deres-Cohen et al., 2021). Another study had 27 sessions from 27 psychotherapies and the last study had 40 sessions from 40 psychotherapies and were both chosen to be rated as case series (Christian et al., 2021). This approach was justified to enable standardization and comparison of results. In general, most studies were somewhat found to be sensitive to risk of bias. Seven studies got fairly good ratings (Avdi and Seikkula, 2019; Negri et al., 2019; Del Giacco et al., 2020; Christian et al., 2021; Deres-Cohen et al., 2021; Luo et al., 2022; Mylona et al., 2022), nine studies got moderate ratings (Tomicic et al., 2015; Morán et al., 2016; Rocco et al., 2017; Zalman et al., 2019; Aafjes-van Doorn and Müller-Frommeyer, 2020; Altimir and Valdés-Sánchez, 2020; Zilcha-Mano et al., 2020; Luo et al., 2021; Mylona and Avdi, 2021), while one study was given a low rating (DiMascio et al., 1957). The most common causes of bias were lack of post-session interventions of clinical conditions, missing descriptions of diagnostic assessments of the patients, and unclear or missing descriptions of inclusion criteria for the cases chosen for analysis.

### 3.4 The association between interpersonal coordination and alliance rupture and repair

Based on the initial assessment of study characteristics, methodology, and quality ratings, the next section provides a description of the findings on the association between rupture repair processes and ICD. Interestingly, the reviewed studies aimed at different pathways for investigating the association, including idiographic analysis (i.e., specific patterns for each session), nomothetic analysis (i.e., patterns across the entire sample), and subgroup analysis (i.e., calculated subgroups baring the same significant characteristics). In the following paragraph, the link between different modalities and rupture and repair processes are presented, including descriptions of these different pathways. A synthesized overview of these finding is provided in Table 2. Significant results are presented if stated in the studies. All figures are presented as published in the original papers.

### 3.5 Interpersonal coordination as a potential marker of alliance rupture repair

Overall, ICD in movement, vocalization, facial expression, biological markers, and physiology were linked to alliance ruptures and repairs in psychotherapy. Some studies directly found ICD to function as marker of rupture and repair processes (n = 4) (Negri et al., 2019; Aafjes-van Doorn and Müller-Frommeyer, 2020; Zilcha-Mano et al., 2020; Deres-Cohen et al., 2021), while other studies identified overall ICD patterns during rupture and repair segments, suggesting that ICD might function as a potential indicator of rupture and repair processes (n = 9) (DiMascio et al., 1957; Morán et al., 2016; Avdi and Seikkula, 2019; Zalman et al., 2019; Altimir and Valdés-Sánchez, 2020; Del Giacco et al., 2020; Christian et al., 2021; Mylona and Avdi, 2021; Mylona et al., 2022). Finally, some studies found more session- or dyadic-specific associations (n = 3) (Tomicic et al., 2015; Luo et al., 2021, 2022). These findings are further elaborated below to specify characteristics regarding the association between rupture repairs and ICD.

Of the studies directly addressing ICD as a marker of ruptures and repairs, one study found non-verbal synchrony to be significantly associated with confrontational ruptures within dyads (state-like) (*B* = 0.19, *SE* = 0.09, *p* = 0.03), but not between dyads (trait-like) over the course of treatment in 75 dyads (Deres-Cohen et al., 2021). In this study, withdrawal ruptures were not found to be significantly associated with non-verbal synchrony either within or across sessions. Another study examining initial intake sessions for 40 dyads found that the patient’s linguistic style in the middle of a session accounted for 54.5% of the alliance score at the final third of the session for a subgroup of four patients [R^2^ = 0.545, *F*(4.35) = 10.46, *p* < 0.001]. This study also identified a pattern of patient and therapist speech characterized by fewer disfluencies in sessions with many ruptures (Negri et al., 2019). Another study found language to positively predict the frequency of ruptures within seven sessions from the same pseudo-dyad (B = 6.78, SE = 2.61, t = 2.59, *p* = 0.007) (Aafjes-van Doorn and Müller-Frommeyer, 2020). Finally, one study found high correlation between the patient’s and the therapist’s changes in oxytocin from pre-sessions to post-sessions over the course of treatment (r = 0.85), and found changes in the levels of oxytocin (OT) from pre- to post sessions in both the patient and the therapist in sessions characterized by multiple ruptures, reflecting oxytocin as a potential biomarker in the state-like alliance during each session (patient’s changes in OT levels: pre-post session 4: −0.11, 8: 0.13, 12: 0.13, and 16: 0.08, and therapist’s changes in OT levels: pre-post session 4: −0.28, 8: −0.01, 12: 0.07, 16: 0.15) (Zilcha-Mano et al., 2020). To exemplify, session 4 was characterized by multiple confrontational (1.96) and withdrawal ruptures (2.96) rated with the 3RS and showed changes for both patient and therapist in the OT levels (patient OT change: 0.08, and therapist OT change: 0.08). The measure was found to be sensitive to both confrontational ruptures and withdrawal ruptures. Furthermore, a measure of oxytocin over the course of treatment showed an overall rise of the patient’s oxytocin level (*r* = −0.11 at the beginning and *r* = 0.08 at the end of treatment) combined with symptom reduction (Hamilton Rating Scale for Depression from 27 at the beginning of treatment to 8 at termination), also indicating that oxytocin might function as a biomarker for more trait-like processes (Zilcha-Mano et al., 2020).

Of the studies suggesting that ICD might serve as an overall indicator of alliance ruptures and repairs, four studies examined physiological arousal (ASV) within sessions, where one of these studies somewhat contrasted the others. Of note, three of these studies were on the same population (Avdi and Seikkula, 2019; Mylona and Avdi, 2021; Mylona et al., 2022). Two of these studies showed similar results (Avdi and Seikkula, 2019; Mylona et al., 2022), while the third was contrasting (Mylona and Avdi, 2021). One of the four studies examined one session from the beginning of treatment and found embodied attunement of both physiological arousal, facial expressions, and postural congruence between patient and therapist to be highest during rupture events in comparison to no-rupture events (Avdi and Seikkula, 2019). Mylona et al. (2022) showed how increased physiological arousal was present during shift from a period of ruptures to the beginning of repairment of the alliance in one session from the end of treatment. Yet, another study examined sessions from the therapy of one dyad and found both higher heart rate of the patient and higher heart rate of the therapist to be associated with higher observer-rated tension scores for the patient during sessions (rank order correlation = −0.58) (DiMascio et al., 1957). The fourth study did not find significant effect of rupture occurrence reflected in the participants physiological arousal when examining the variables rupture; no rupture in 12 sessions from seven dyads (Mylona and Avdi, 2021). Nevertheless, this study found that a specific rupture type—mixed ruptures—was associated with increased client arousal during periods with multiple ruptures (intercept = 18.96, *t*(34665) = 11.55, *p* < 0.0005). No association was found in relation to withdrawal ruptures, while confrontation ruptures were associated with lower client arousal compared to no rupture segments (intercept = −2.78, *t*(34665) = −2.53, *p* < 0.005). One of the four studies was found to be specifically prone to risk of bias, which limits the reliability of the findings (DiMascio et al., 1957). Yet, these results indicate that even within the same modality, with data from the same data pool, differentiation can be present as to how physiological arousal is related to ruptures and repairs and do not point in any conclusive direction regarding physiological arousal as an indicator of rupture and repair episodes.

Three studies examined facial-affective expressions. One of these studies made a qualitative analysis of facial expressions as supplement to content analysis (Avdi and Seikkula, 2019), while two studies applied the Facial Action Coding System (Morán et al., 2016; Altimir and Valdés-Sánchez, 2020). Both studies using the Facial Action Coding System found higher probabilities of the patients gazing away from the therapists during rupture episodes. Contrasting to each other, one of these studies found rupture episodes to be associated with the patient showing more negative emotions (hierarchical linear modeling fixed effect modeling; anger: OR = 0.29, *p* < 0.05, fear: OR = 0.00, *p* < 0.01, control of facial expression OR = 0.22, *p* < 0.05) (Altimir and Valdés-Sánchez, 2020), while the other study found rupture episodes to be associated with the patient showing more positive emotions (55.7% presence of regulatory facial behavior) (Morán et al., 2016). One of the two studies also found the therapist’s increased probability of gazing at the patient and displaying self-soothing and regulatory control behaviors during rupture events (Altimir and Valdés-Sánchez, 2020), while the other study found rupture episodes to be associated with the therapist’s increased use of adapters (defined as contacts of one part of the body or face with another part of the face) (Morán et al., 2016). Only one of the two studies examined facial characteristics of resolution processes and found a consistent pattern, that in moments of resolution the patient’s facial behavior was characterized by gazing at the therapist and displaying self-soothing behavior (OR = 4.91, *p* < 0.05 and OR = 7.57, *p* < 0.01, respectively). The therapist was more likely to display so called illustrators, manifested by lifting inner and outer eye brows (OR = 3.51, *p* < 0.05) (Altimir and Valdés-Sánchez, 2020). All three studies demonstrated specific facial characteristics during rupture events within sessions, but like with physiological arousal, the results suggest that within the same modality association characteristics might vary.

Two studies examined how specific patterns of in-session behaviors of warmth and dominance between patient and therapist contributed to, or resulted from, ruptures. Both studies found high heterogeneity in the association between interpersonal behaviors and ruptures varying from session to session and between dyads. One of the studies identified distinct interpersonal patterns associated with ruptures for three different dyads (Luo et al., 2021). The other study did not find any overall associations between ruptures and ICD at a nomothetic level, but identified a subgroup of two sessions within the same dyad where higher synchronization on dominance was associated with confrontational ruptures (Luo et al., 2022). Also on an idiographic level, this study identified multiple session-specific associations. On a subgroup level of seven sessions, this study found a pattern where the therapist’s dominance negatively predicted concurrent withdrawal ruptures (Luo et al., 2022). Both studies was rated a fairly good quality study according to risk of ruptures within sessions.

### 3.6 Functionalities of ICD during rupture repair episodes

Besides examining ICD as a potential marker of rupture repairs, several studies also investigated ICD’s potential role during such moments of interaction. See Table 2 for an overview of these findings. Overall, ICD was found to be related to emotion regulation (n = 13) (Tomicic et al., 2015; Morán et al., 2016; Rocco et al., 2017; Avdi and Seikkula, 2019; Negri et al., 2019; Zalman et al., 2019; Altimir and Valdés-Sánchez, 2020; Del Giacco et al., 2020; Christian et al., 2021; Luo et al., 2021, 2022; Mylona and Avdi, 2021; Mylona et al., 2022), meaning-making (n = 5) (Tomicic et al., 2015; Morán et al., 2016; Rocco et al., 2017; Avdi and Seikkula, 2019; Negri et al., 2019; Mylona et al., 2022), empathy (n = 1) (Mylona et al., 2022), trust (n = 2) (Avdi and Seikkula, 2019; Negri et al., 2019), warmth (n = 1) (Negri et al., 2019), safety (n = 1), and affiliation (n = 1) (Mylona et al., 2022). Several findings demonstrated how different modalities functioned as a complex dynamic whole, allowing mutual regulation and shaping each other during rupture and repair episodes in the early reciprocal alliance construction (Del Giacco et al., 2020), within sessions (Avdi and Seikkula, 2019; Mylona et al., 2022), and over the course of treatment (Tomicic et al., 2015; Morán et al., 2016). Various studies also found different modalities to be dependent on each other in the reciprocal co-regulation process during rupture and repair episodes (Tomicic et al., 2015; Altimir and Valdés-Sánchez, 2020; Del Giacco et al., 2020). Lastly, one study found ICD to be a moderating factor of positive alliance formation (Rocco et al., 2017).

### 3.7 Outcome

Besides looking at the association between rupture repairs and ICD, several studies reported outcome information (n = 7). To determine outcome, all these studies measured outcome with different pre-post client self-reported questionnaires including the Clinical Outcomes in Routine Evaluation-Outcome Measure (Evans et al., 2002) (n = 2) (Avdi and Seikkula, 2019; Mylona et al., 2022), Hamilton Rating Scale for Depression (Hamilton, 1960) (n = 1) (Zilcha-Mano et al., 2020), Outcome Questionaire (Lambert et al., 1996) (n = 3) (Tomicic et al., 2015; Morán et al., 2016; Altimir and Valdés-Sánchez, 2020), Core Conflictual Relationship Theme (Luborsky et al., 1994) (n = 1) (Rocco et al., 2017), Symptom Check List (SCL-90 R) (Derogatis and Melisaratos, 1983) (n = 1) (Rocco et al., 2017), and The Hierarchy of Generic Change Indicators (Krause et al., 2007) (n = 1) (Altimir and Valdés-Sánchez, 2020). Three studies examined good outcome cases (Avdi and Seikkula, 2019; Zilcha-Mano et al., 2020; Mylona et al., 2022), three studies examined both good and poor outcome cases (Tomicic et al., 2015; Morán et al., 2016; Rocco et al., 2017), and one study examined a case with no clinically significant change (Altimir and Valdés-Sánchez, 2020). Only one study directly related ICD and alliance rupture and repair processes to the outcome of treatment, comparing a good outcome and bad outcome case. This study found higher attunement levels and coordination of speech rate (Verbal Attunement Index (VAI); *F* = 16.043, *p* = 0.000), and almost higher coordination in rupture and repair events in a good outcome case compared to a poor outcome case (Session Quality Index (SQI); *F* = 3.421, *p* = 0.07) (Rocco et al., 2017). The other six studies stated the use of pre-post outcome measures as contextual frameworks for their case presentations.

## 4 Discussion

The aim of this systematic review was to collect and synthesize findings examining the link between ICD and alliance rupture and repair processes to expand our understanding of the underlying mechanisms in constructing, maintaining, negotiating, and repairing the therapeutic alliance. In the 17 studies identified, results suggest that coordination in several modalities of movement, vocalization, facial expression, biology, and physiology was linked to alliance ruptures and repairs in psychotherapy. Furthermore, ICD was identified as a potential marker of alliance rupture and repair episodes and was found to play a crucial role in the mutual affective regulation between patient and therapist during such events.

### 4.1 Key findings

A key finding in the reviewed studies is the fact that results indicate that ICD might be implicated in rupture and repair episodes in relation to emotional regulation (Tomicic et al., 2015; Morán et al., 2016; Rocco et al., 2017; Avdi and Seikkula, 2019; Negri et al., 2019; Zalman et al., 2019; Altimir and Valdés-Sánchez, 2020; Del Giacco et al., 2020; Christian et al., 2021; Luo et al., 2021, 2022; Mylona and Avdi, 2021; Mylona et al., 2022), empathy (Mylona et al., 2022), trust (Avdi and Seikkula, 2019; Christian et al., 2021), meaning-making (Tomicic et al., 2015; Morán et al., 2016; Rocco et al., 2017; Avdi and Seikkula, 2019; Negri et al., 2019; Mylona et al., 2022), warmth (Negri et al., 2019), and provision of safety (Negri et al., 2019). In general, this is in line with previous alliance research, which has identified ICD to be related to empathy (Marci and Orr, 2006), attachment security (Diamond and Fagundes, 2010), stress contagion (Waters et al., 2014), and emotional security and regulation of emotional distress (Feldman, 2007; Timmons et al., 2015). However, inconsistencies have been identified within the field of ICD, and some studies have not found associations between synchrony in the therapeutic interaction and empathy (Gaume et al., 2019). For instance, one study found pitch synchrony to be negatively associated with therapist empathy (Reich et al., 2014). Thus, even though most studies support the link between synchronization and different functionalities in the therapeutic interaction and the alliance, the literature is inconsistent about the optimal level of coordination. While higher interpersonal coordination has repeatedly been shown to be associated with positive alliance and outcome (Ramseyer and Tschacher, 2016; Rocco et al., 2017), some studies suggest that too much attunement may have a negative impact on the patient’s ability to self-regulate emotions on their own, as the patient could come to rely too much on the therapist in the process to regulate their inner states (Galbusera et al., 2019). Several studies suggest upholding a healthy homeostatic balance in the process to enlarge the establishment and enhancement of the patient’s capacity to regulate their own emotional states and feelings of safety (Reed et al., 2015; Kleinbub et al., 2020). Depending on the context, pathology, and treatment type, no coordination, misattunement, or deliberately causing tension to create spaces of possibilities during sessions might be favorable to enable corrective experiences in the management of critical situations (Colli and Lingiardi, 2009). In the reviewed findings, only one study found higher attunement during rupture repair episodes to be associated with positive outcome (Rocco et al., 2017). Even though no conclusions can be drawn from these initial findings yet, the results suggest that the therapist and patient coordinate their responses during rupture repair episodes.

Another important finding in the reviewed studies was the identification of ICD as a potential predictor or marker of rupture and repair episodes mainly within (Deres-Cohen et al., 2021), but in some cases also over the course of treatment (Zilcha-Mano et al., 2020). Among the specific findings, results suggest that non-verbal synchrony might serve as a marker of confrontational ruptures within sessions (Deres-Cohen et al., 2021), heart rate could be a physiological marker of tension within sessions (DiMascio et al., 1957; Zalman et al., 2019), and oxytocin might function as a biomarker for both confrontational and withdrawal ruptures within sessions and over the course of treatment, the latter represented through an increase in OT levels pre-sessions combined with higher alliance ratings, and self-reported symptom reduction (Zilcha-Mano et al., 2020). Also, different kinds of vocalization could be a predictor of the ruptures within sessions (Negri et al., 2019; Aafjes-van Doorn and Müller-Frommeyer, 2020; Christian et al., 2021). Similar results have been identified in other studies that also identified ICD to reflect rupture episodes (Zilcha-Mano et al., 2018; Dolev-Amit et al., 2022). While these two studies are not included in this review due to lack of therapist measures, they no doubt support the notion of ICD as a potential marker of alliance rupture and repair processes. In addition, a rising number of studies have argued that higher attunement could function as an indicator of unfavorable processes in the psychotherapeutic interaction, including premature drop out, lack of progress, increased symptomatology, conflicts, and disagreements (Friedman, 2020; Ramseyer, 2020; Coutinho et al., 2021). These findings may help increase understanding of divergent results in research regarding the association between ICD and the alliance, where some studies have found a positive correlation (Ramseyer and Tschacher, 2011; Tal et al., 2023), while others have not (Paulick et al., 2018a; Ramseyer, 2020). Thus, these results have the potential to explain the mechanisms underlying the associations between ICD and the alliance by showing how different modalities may be linked to confrontational and/or withdrawal ruptures. Notably, the reviewed findings included primarily state-like examinations of within session analyses looking at questions about how to identify and address temporary fluctuations in the moment–to–moment interpersonal interaction within sessions as the sessions unfold. Only a few studies included trait-like associations between ICD and rupture repair processes, which are statistically and conceptually different from the state-like examinations as they tend to represent more stable and enduring patterns, which can serve as important predispositions to certain states (Falkenström and Larsson, 2017; Zilcha-Mano and Errázuriz, 2017; Rubel et al., 2018). Though these results are promising, they do not allow any generalizable assumptions, but strongly endorse future work to further our knowledge concerning if and how different modalities might serve as potential markers of significant events in the interpersonal context of psychotherapy, both in relation to state-like and trait-like components. Furthermore, even though the reviewed findings highlight the beneficial potential of studying the link between ICD and rupture repair episodes, the results also reveal that a simple linkage between ICD and rupture repair does not appear to be sufficient. Findings varied significantly in relation to how alliance rupture and repair were associated with ICD. Several quite divergent findings were identified and are discussed in the next sections.

### 4.2 Divergent findings across and within modalities

As already illustrated, evidence is unclear about the effects of different modalities (Scheidt et al., 2021). Different modalities are suggested to reflect different aspects of the therapeutic alliance (Schoenherr et al., 2019; Altmann et al., 2021; Scheidt et al., 2021). In the reviewed findings, variations were identified in different modalities and their associations with the variables rupture–no rupture and with specific rupture types. Among the results, one study foun increased non-verbal synchrony between patient and therapist to correspond with confrontational ruptures within sessions (Deres-Cohen et al., 2021), while lower physiological arousal in the patient was associated with confrontational ruptures within session in another study (Mylona and Avdi, 2021). Also, non-verbal synchrony was found to be associated with confrontational ruptures but not withdrawal ruptures on a state-like level but not a trait-like level (Deres-Cohen et al., 2021), while another study found indication that oxytocin is sensitive to both confrontational and withdrawal ruptures, both on a state-like and trait-like level (Zilcha-Mano et al., 2020). These findings imply that different modalities might be sensitive to different rupture and repair processes. Previous research has proposed that various modalities most likely follow different time scales within and across sessions, which may lead to unique time dynamics in each modality in relation to rupture and repairs (Kykyri et al., 2019; Scheidt et al., 2021). Thus, association characteristics might very likely be highly dependent on the measured response (Helm et al., 2014; Kleinbub et al., 2020). While previous studies on ICD have, to at large degree, focused on primarily one modality, the integration of multiple modalities in psychotherapy research is still in its infancy. An interesting future question is to elaborate further on if and how different modalities are connected to each other during rupture and repairs events (Noy et al., 2015; Zhang et al., 2016; Koole et al., 2020; Scheidt et al., 2021; Tourunen et al., 2022).

Besides locating heterogeneity across modalities heterogeneity was also identified within the same modalities. For example, rupture episodes were both linked to facial-affective expressions of the patient showing more negative emotions in one study (Altimir and Valdés-Sánchez, 2020), and the patient showing more positive emotions in another study (Morán et al., 2016). Moreover, a third study of facial-affective expressions, not included in this review due to lack of therapist measures, found joy, positive emotional valence, and social smile to correspond with withdrawal ruptures and patient absence of emotional valence to correspond with confrontational ruptures (Barros et al., 2016). Similar discrepancies were identified in relation to physiological arousal (Avdi and Seikkula, 2019; Mylona and Avdi, 2021; Mylona et al., 2022). The examination of ICD in psychotherapy is fragmented in relation to study designs, and methodological and analytical approaches (Kleinbub, 2017), and it is possible that these findings reflect differences in methodology, settings, and samples between the studies. For instance, Morán et al. (2016) and Altimir and Valdés-Sánchez (2020) did not account for comparison between specific rupture types. A comparison between confrontational ruptures and withdrawal ruptures might have exposed other patterns of facial-affective behavior as seen in Barros et al. (2016).

As proposed in previous research it is also possible that association characteristics might be more dyadic- or session-specific (Barlow and Nock, 2009; Constantino et al., 2020). As the above mentioned studies only examined either a few dyads (Barros et al., 2016; Morán et al., 2016), one dyad (Altimir and Valdés-Sánchez, 2020), or a single session (Avdi and Seikkula, 2019; Mylona et al., 2022) no conclusions can be drawn. However, two studies in the reviewed findings support the notion of more dyadic- or session-specific associations (Luo et al., 2021, 2022). They identified several session-specific associations where no dyads or even sessions shared precisely the same dynamics. These results might suggest that association characteristics are highly context-dependent and emphasize the importance of session-specific adjustments when facing ruptures during the therapeutic interaction.

### 4.3 Mental distress and type of therapy

As the main aim of this review was to examine the association between ICD and rupture repair processes, and as this area is still in its infancy, this review included a broad population receiving different types of psychotherapy. As such, there has not been accounted for the possibility that associations between ICD and rupture repair may vary depending on the type of personal issues, mental distress, diagnosis or therapy type, which has previously been shown (Kupper et al., 2015; Paulick et al., 2018b; Bar-Kalifa et al., 2019). Additionally, one study in the reviewed findings included a non-clinical group (Negri et al., 2019). Even as this study aimed at examining mental distress, this study was chosen for inclusion, as it primarily examined people suffering from different disorders. Moreover, it also provided a calculation of differentiation related to rupture–no rupture segments, and did not find any differences between diagnoses or between the non-clinical sample and a clinical sample (Negri et al., 2019). However, it is possible that other associations might have been revealed if differentiation between diagnosis or therapy type had been conducted. None of the studies examined differentiation between therapy type and rupture–no rupture events. As such, the varied population and inclusion of different therapy types limit the generalizability of the findings. However, as ICD is thought to be more related to common factors than to a specific diagnosis or method, the findings might be relevant to psychotherapy in general (Wiltshire et al., 2020). Because the research on ICD and rupture repair is still scarce, it was found premature to conduct a comprehensive synthesis of diagnosis-specific associations and treatment type. However, the potential of including these foci in future studies could be of great importance, as for instance psychomotor disturbances related to different diagnoses might have significant influence on patient ability to coordinate and engage in the therapeutic interaction (Paulick et al., 2018b).

### 4.4 Quality ratings

The results in this review are based on 17 studies including a limited number of people (N = 185), ranging from one patient to 75 patients, where some studies only examined single sessions (Rocco et al., 2017; Mylona et al., 2022). The results revealed that most studies were somewhat prone to selection bias, as well as were missing outcome ratings. Moreover, high heterogeneity was identified in assessment strategies, study design, methods, and quantification of results hindering interpretation, comparison of results, and pooling of association estimates. Future work should indeed focus on decreasing risk of bias, paying special attention to selection bias and outcome to increase the validity of the results.

### 4.5 Clinical implications

Despite the limitations in the reviewed studies, several main opportunities of these initial findings are relevant to examine further in the future. For instance, the possibility to develop and adapt real-time in-session feedback systems to the therapist on rupture markers. Such a system could support the use of timely and contingent interventions and support management, emotion regulation, and meaning-making during challenging events beyond conversational content during sessions (Safran, 2003), and might help prevent deterioration and premature drop out (Eubanks et al., 2018; Wiltshire et al., 2020; Deres-Cohen et al., 2021). The knowledge could be integrated into our theoretical models to outline more explicit intervention strategies for the more implicit processes during therapeutic interaction, which could be integrated into the training and supervision of psychotherapists (Kleinbub et al., 2020; Jensen et al., 2021). Nevertheless, it remains unclear to what degree clinicians are able to consciously adapt interpersonal coordination strategies into their clinical models and what the gains of this would be which is still a question to be answered in the future (Kleinbub, 2017).

### 4.6 Limitations and future recommendations

The ICD paradigm in adult psychotherapy has provided new and innovative ways to broaden our understanding of the interaction between patient and therapist (Atzil-Slonim and Tschacher, 2020). Automated methods and technologies offer new and time-saving approaches. However, instruments vary largely, and the heterogeneity in the instruments and study designs in cumbers comparability and interpretation of the findings. Even though most of the studies used 3RS, variation was observed regarding how data was processed. Studies differed regarding which variables they examined. Some investigated the variable rupture–no rupture, while other studies examined specific rupture types. Also, some studies rated 5-min segments within sessions while others rated 30-s intervals, and finally some rated from the first hint of the rupture to the first hint of resolution or rated for intensity of the ruptures. Besides 3RS, other rupture and repair instruments were used, including SWAO (Berk, 2013), CIS (Colli and Lingiardi, 2009), CIS-R (Colli et al., 2019), and interaction process analysis (Bales, 1970). There is evidence that the prevalence of ruptures and repairs differs significantly depending on the method used for assessing them (Strauss et al., 2006; Sommerfeld et al., 2008; Eubanks et al., 2018). Evidently, different procedures probably lead to inconsistent findings. This calls for further examination and application of more homogeneous methods and procedures to enable meaningful generalizability, comparison, and exploration of contrasting findings. A strength in the reviewed findings was that most of the included studies used direct observer-based ratings, and were therefore more likely to have a higher sensitivity in rupture identification than if they had applied indirect rupture assessment tools found to be associated with underreporting of rupture episodes (Coutinho et al., 2014b). The direct assessments were found to reveal detailed descriptions of the interactional dynamics during rupture repair events, which was of particular interest in this review (Bennett et al., 2006; Aspland et al., 2008). Unfortunately, direct assessment is time- and cost-intensive, which probably reflects the small sample sizes included in this review (Koole and Tschacher, 2016). Another methodological shortcoming is that the version of 3RS (2015) applied in most of the included studies only focuses on the patient’s contributions to ruptures and on the therapist’s contributions to resolution. This could limit identification of associations between ICD and ruptures initiated by the therapist and associations between ICD and resolution initiated by the patient. Future studies should include measures taking both the patient’s and therapist’s mutual contribution into account (i.e., the updated version of 3RS, 2022). Finally, several studies purposely selected sessions with high levels of ruptures to maximize the variation in rupture–no rupture segments. Though it is possible that ICD manifestation varies in dyads or sessions with milder expressions.

As 3RS, the assessment of ICD also revealed high heterogeneity. Studying ICD’s association to alliance processes is highly complex and includes several methodological challenges. One challenge in comparing results in the included studies was that they differed in either quantifying ICD results separately for the patient and the therapist (Christian et al., 2021; Mylona and Avdi, 2021) or used synchronization measures between patient and therapist modalities (Zilcha-Mano et al., 2020; Deres-Cohen et al., 2021). While alliance rupture and repair episodes are interactional processes where the response of one interacting partner affects the other in mutual co-regulation, future studies are recommended to explore the mutual interactional process by integrating models capturing more of the interactional process than done by studying the two partners separately (Scheidt et al., 2021). Thus, several of the included studies incorporated methods, for instance using measurements of lagged or windowed procedures, to provide evidence of moment-to-moment attunement, as well as misattunement, between patient and therapist (Deres-Cohen et al., 2021; Luo et al., 2021, 2022; Mylona et al., 2022). Additionally, more complex quantification methods were applied to examine the dependence and interplay between verbal and non-verbal components during rupture repair episodes, including DSEM (Luo et al., 2021), GIMME (Luo et al., 2022), and a polar coordinate analysis (Del Giacco et al., 2020), which should be elaborated further in future work. Another shortcoming is the lack of examination of coordination in relation to pseudo-synchronization, understood as the examination of synchronization caused by coincidence, to obtain the strength of the coordination between patient and therapist, for instance as shown in the included study of Deres-Cohen et al. (2021). Future work can advantageously ensure that the coordination identified in each modality is related to more than chance (F. Ramseyer and Tschacher, 2010; Scheidt et al., 2021). Finally, the measures of ICD were derived in different ways, either as automated computer-based assessments (Deres-Cohen et al., 2021; Mylona and Avdi, 2021; Mylona et al., 2022) or manual data collection performed by raters (Zalman et al., 2019; Altimir and Valdés-Sánchez, 2020). As manual data processing is time-consuming and costly, research needs to further the development and integration of more computer-based assessment tools to make it easier to adapt these methods in the study of therapeutic interaction in larger sample sizes and to be able to integrate perspectives on temporal variations over the course of treatment more efficiently.

## 5 Conclusion

Overall, the reviewed findings underscore the value of combining different approaches to further our understanding of the underlying mechanisms of rupture and repair processes in the therapeutic interaction. While the association between ICD and alliance ruptures and repairs to a high degree remains inconsistent, the reviewed findings suggest that different modalities of physiology, movement, biomarkers, linguistics, language, and voice are linked to alliance rupture and repair processes in psychotherapy. Moreover, ICD was shown to serve central functionalities in relation to mutual emotion regulation, empathic response, trust, safety, and meaning-making during alliance rupture and repair episodes. Most studies were found to be prone to risk of bias, and future work should focus on decreasing selection bias, include larger sample sizes, and include outcome measures. Nevertheless, by examining and raising awareness about the unconscious and subconscious processes in the dyadic interplay in psychotherapy, our hope is that we have sensitized practitioners and trainees to the importance of the intracorporeal dynamics beyond the verbal aspects, which in future work could allow for a richer model of psychotherapeutic interaction and practice (Fonagy and Target, 2007; Fuchs, 2017). Furthermore, results show that automated methods may have the potential to translate intuitive processes into measurable factors, which could have great impact for researchers, clinicians, and supervisors. We hope that these initial findings will be expanded in future work.

## Author contributions

SH: Writing–original draft, Writing–review and editing. MK: Supervision, Writing–review and editing. GT: Supervision, Writing–review and editing.
