# Supplementary material for: The role of interpersonal coordination dynamics in alliance rupture and repair processes in psychotherapy—A systematic review
Source: Front Psychol. 2024 Jan 4;14:1291155. doi: 10.3389/fpsyg.2023.1291155 (PMC10794593; doi:10.3389/fpsyg.2023.1291155)
Supplement: Supplementary file 1 [file Table_1.DOCX]

Supplementary Material

# Supplementary Figures and Tables

Appendix 1

### Databases and results

| Database | Platform | Results | Date |
| --- | --- | --- | --- |
| PubMed | PubMed.gov | 418 | 02.06.2023 |
| Embase | Embase.com | 518 | 02.06.2023 |
| PsycINFO | APA | 589 | 02.06.2023 |
| Scopus | Scopus.com | 764 | 02.06.2023 |
| All  ÷ duplicates with EndNote |  | 2289  1262 |  |

### PubMed

| Search | Query |
| --- | --- |
| #5 | Search: **((("Professional-Patient Relations"[MeSH Terms] OR "relation*"[Text Word] OR "alliance*"[Text Word] OR "interaction*"[Text Word] OR "interpersonal*"[Text Word] OR "Psychotherapeutic Processes"[Mesh]) AND (therapist*[Text Word] OR psychotherapist*[Text Word] OR psychologist*[Text Word] OR psychiatrist*[Text Word] OR analyst[Text Word] OR ((clinician*[Text Word] OR session[Text Word]) AND (psych*[Text Word] OR mental health[Text Word])))) AND (("rupture*"[Text Word] OR "repair*"[Text Word] OR "tension*"[Text Word] OR "resolution*"[Text Word] OR "resolve*"[Text Word] OR "confrontat*"[Text Word] OR "Negotiating"[MeSH Terms] OR "negotiat*"[Text Word] OR "conflict*"[Text Word]) OR (deterioration*[Text Word]))) AND (((("synchron*"[Text Word] OR "coordinat*"[Text Word] OR "covariation*"[Text Word] OR "coheren*"[Text Word] OR "collu*"[Text Word] OR "linkage*"[Text Word] OR "contagion*"[Text Word] OR "attun*"[Text Word] OR "align*"[Text Word] OR "concordance*"[Text Word] OR "mirror*"[Text Word]) OR ("Nonverbal Communication"[Mesh])) OR ("Galvanic Skin Response"[Mesh])) OR (language style[Text Word] OR facial[Text Word] OR skin conductance[Text Word] OR skin temperature*[Text Word] OR respirati*[Text Word] OR heart rate[Text Word] OR speech rate[Text Word] OR vocal[Text Word] OR pitch[Text Word] OR nonverbal[Text Word] OR nonverbal[Text Word] OR body movement[Text Word] OR body language*[Text Word] OR EDA[Text Word] OR vocalizat*[Text Word] OR gestur*[Text Word] OR acoustic*[Text Word] OR dermal*[Text Word] OR autonomic nervous system[Text Word] OR physiological arousal[Text Word] OR biomarker*[Text Word]))** Sort by: **Publication Date** |
| #4 | Search: **((("synchron*"[Text Word] OR "coordinat*"[Text Word] OR "covariation*"[Text Word] OR "coheren*"[Text Word] OR "collu*"[Text Word] OR "linkage*"[Text Word] OR "contagion*"[Text Word] OR "attun*"[Text Word] OR "align*"[Text Word] OR "concordance*"[Text Word] OR "mirror*"[Text Word]) OR ("Nonverbal Communication"[Mesh])) OR ("Galvanic Skin Response"[Mesh])) OR (language style[Text Word] OR facial[Text Word] OR skin conductance[Text Word] OR skin temperature*[Text Word] OR respirati*[Text Word] OR heart rate[Text Word] OR speech rate[Text Word] OR vocal[Text Word] OR pitch[Text Word] OR nonverbal[Text Word] OR nonverbal[Text Word] OR body movement[Text Word] OR body language*[Text Word] OR EDA[Text Word] OR vocalizat*[Text Word] OR gestur*[Text Word] OR acoustic*[Text Word] OR dermal*[Text Word] OR autonomic nervous system[Text Word] OR physiological arousal[Text Word] OR biomarker*[Text Word])** Sort by: **Publication Date** |
| #3 | Search: **("rupture*"[Text Word] OR "repair*"[Text Word] OR "tension*"[Text Word] OR "resolution*"[Text Word] OR "resolve*"[Text Word] OR "confrontat*"[Text Word] OR "Negotiating"[MeSH Terms] OR "negotiat*"[Text Word] OR "conflict*"[Text Word]) OR (deterioration*[Text Word])** Sort by: **Publication Date** |
| #2 | Search: **therapist*[Text Word] OR psychotherapist*[Text Word] OR psychologist*[Text Word] OR psychiatrist*[Text Word] OR analyst[Text Word] OR ((clinician*[Text Word] OR session[Text Word]) AND (psych*[Text Word] OR mental health[Text Word]))** Sort by: **Publication Date** |
| #1 | Search: **"Professional-Patient Relations"[MeSH Terms] OR "relation*"[Text Word] OR "alliance*"[Text Word] OR "interaction*"[Text Word] OR "interpersonal*"[Text Word] OR "Psychotherapeutic Processes"[Mesh]** Sort by: **Publication Date** |

### Embase

| No. | Query | Results |
| --- | --- | --- |
| #14 | #1 AND #5 AND #8 AND #13 | 518 |
| #13 | #9 OR #10 OR #11 OR #12 | 2576616 |
| #12 | 'language style':ti,ab,kw OR facial:ti,ab,kw OR 'skin conductance':ti,ab,kw OR 'skin temperature*':ti,ab,kw OR respirati*:ti,ab,kw OR 'heart rate':ti,ab,kw OR 'speech rate':ti,ab,kw OR vocal:ti,ab,kw OR pitch:ti,ab,kw OR nonverbal:ti,ab,kw OR 'nonverbal':ti,ab,kw OR 'body movement':ti,ab,kw OR 'body language*':ti,ab,kw OR eda:ti,ab,kw OR vocalizat*:ti,ab,kw OR gestur*:ti,ab,kw OR acoustic*:ti,ab,kw OR dermal*:ti,ab,kw OR 'autonomic nervous system':ti,ab,kw OR 'physiological arousal':ti,ab,kw OR biomarker*:ti,ab,kw | 1435182 |
| #11 | 'electrodermal response'/de | 8101 |
| #10 | 'nonverbal communication'/exp | 54486 |
| #9 | synchron*:ti,ab,kw OR coordinat*:ti,ab,kw OR covariation*:ti,ab,kw OR coheren*:ti,ab,kw OR linkage*:ti,ab,kw OR contagion*:ti,ab,kw OR attun*:ti,ab,kw OR align*:ti,ab,kw OR concordance*:ti,ab,kw OR mirror*:ti,ab,kw | 1161473 |
| #8 | #6 OR #7 | 2093469 |
| #7 | rupture*:ti,ab,kw OR repair*:ti,ab,kw OR tension*:ti,ab,kw OR resolution*:ti,ab,kw OR resolve*:ti,ab,kw OR confrontat*:ti,ab,kw OR negotiat*:ti,ab,kw OR conflict*:ti,ab,kw OR deterioration*:ti,ab,kw | 2093387 |
| #6 | 'negotiation'/de | 1177 |
| #5 | #2 OR #3 OR #4 | 225623 |
| #4 | (clinician*:ti,ab,kw OR session:ti,ab,kw) AND (psych*:ti,ab,kw OR 'mental health':ti,ab,kw) | 80171 |
| #3 | therapist*:ti,ab,kw OR psychotherapist*:ti,ab,kw OR psychologist*:ti,ab,kw OR psychiatrist*:ti,ab,kw OR analyst:ti,ab,kw | 154995 |
| #2 | 'psychotherapist'/de | 7491 |
| #1 | 'professional-patient relationship'/exp OR relation*:ti,ab,kw OR alliance*:ti,ab,kw OR interaction*:ti,ab,kw OR interpersonal*:ti,ab,kw | 4550177 |

### PsycINFO

| # | Search | Results |
| --- | --- | --- |
| Item 1 | Results for ((((**title**: (therapist*)) *OR* (**title**: (psychotherapist*)) *OR* (**title**: (psychologist*)) *OR* (**title**: (psychiatrist*)) *OR* (**title**: (analyst))) *OR* ((**IndexTermsFilt**: ("Therapists")) *OR* (**IndexTermsFilt**: ("Clinicians")) *OR* (**IndexTermsFilt**: ("Physicians")) *OR* (**IndexTermsFilt**: ("Psychiatrists")) *OR* (**IndexTermsFilt**: ("Clinical Psychologists")) *OR* (**IndexTermsFilt**: ("Counseling Psychologists"))) *OR* ((**abstract**: (therapist*)) *OR* (**abstract**: (psychotherapist*)) *OR* (**abstract**: (psychologist*)) *OR* (**abstract**: (psychiatrist*)) *OR* (**abstract**: (analyst))) *OR* ((**KEYWORDS**: (therapist*)) *OR* (**KEYWORDS**: (psychotherapist*)) *OR* (**KEYWORDS**: (psychologist*)) *OR* (**KEYWORDS**: (psychiatrist*)) *OR* (**KEYWORDS**: (analyst))))) *AND* ((((**IndexTermsFilt**: ("Negotiation")) *OR* (**IndexTermsFilt**: ("Bargaining")) *OR* (**IndexTermsFilt**: ("Conflict Resolution")) *OR* (**IndexTermsFilt**: ("Mediation")))) *OR* (((**title**: (rupture*)) *OR* (**title**: (repair*)) *OR* (**title**: (tension*)) *OR* (**title**: (resolution*)) *OR* (**title**: (resolve*)) *OR* (**title**: (confrontat*)) *OR* (**title**: (negotiat*)) *OR* (**title**: (deterioration*)) *OR* (**title**: (conflict*))) *OR* ((**abstract**: (rupture*)) *OR* (**abstract**: (repair*)) *OR* (**abstract**: (tension*)) *OR* (**abstract**: (resolution*)) *OR* (**abstract**: (resolve*)) *OR* (**abstract**: (confrontat*)) *OR* (**abstract**: (negotiat*)) *OR* (**abstract**: (deterioration*)) *OR* (**abstract**: (conflict*))) *OR* ((**KEYWORDS**: (rupture*)) *OR* (**KEYWORDS**: (repair*)) *OR* (**KEYWORDS**: (tension*)) *OR* (**KEYWORDS**: (resolution*)) *OR* (**KEYWORDS**: (resolve*)) *OR* (**KEYWORDS**: (confrontat*)) *OR* (**KEYWORDS**: (negotiat*)) *OR* (**KEYWORDS**: (deterioration*)) *OR* (**KEYWORDS**: (conflict*))))) *AND* ((((((**IndexTermsFilt**: ("Mirroring"))))) *OR* ((((**title**: (mirror*))) *OR* ((**title**: (concordance*))) *OR* ((**title**: (align*))) *OR* ((**title**: (attun*))) *OR* ((**title**: (contagion*))) *OR* ((**title**: (linkage*))) *OR* ((**title**: (coheren*))) *OR* ((**title**: (covariation*))) *OR* ((**title**: (coordinat*))) *OR* ((**title**: (collu*))) *OR* ((**title**: (synchron*)))) *OR* (((**abstract**: (mirror*))) *OR* ((**abstract**: (concordance*))) *OR* ((**abstract**: (align*))) *OR* ((**abstract**: (attun*))) *OR* ((**abstract**: (contagion*))) *OR* ((**abstract**: (linkage*))) *OR* ((**abstract**: (coheren*))) *OR* ((**abstract**: (covariation*))) *OR* ((**abstract**: (coordinat*))) *OR* ((**abstract**: (collu*))) *OR* ((**abstract**: (synchron*)))) *OR* (((**KEYWORDS**: (mirror*))) *OR* ((**KEYWORDS**: (concordance*))) *OR* ((**KEYWORDS**: (align*))) *OR* ((**KEYWORDS**: (attun*))) *OR* ((**KEYWORDS**: (contagion*))) *OR* ((**KEYWORDS**: (linkage*))) *OR* ((**KEYWORDS**: (coheren*))) *OR* ((**KEYWORDS**: (covariation*))) *OR* ((**KEYWORDS**: (coordinat*))) *OR* ((**KEYWORDS**: (collu*))) *OR* ((**KEYWORDS**: (synchron*)))))) *OR* (((**IndexTermsFilt**: ("Nonverbal Communication")) *OR* (**IndexTermsFilt**: ("Body Language")) *OR* (**IndexTermsFilt**: ("Eye Contact")) *OR* (**IndexTermsFilt**: ("Facial Expressions")) *OR* (**IndexTermsFilt**: ("Gestures")) *OR* (**IndexTermsFilt**: ("Manual Communication")) *OR* (**IndexTermsFilt**: ("Galvanic Skin Response")))) *OR* (((**title**: ("language style")) *OR* (**title**: (facial)) *OR* (**title**: ("skin conductance")) *OR* (**title**: ("skin temperature*")) *OR* (**title**: (respirati*)) *OR* (**title**: ("heart rate")) *OR* (**title**: ("speech rate")) *OR* (**title**: (vocal)) *OR* (**title**: (pitch)) *OR* (**title**: (nonverbal)) *OR* (**title**: ("nonverbal")) *OR* (**title**: ("body movement")) *OR* (**title**: ("body language*")) *OR* (**title**: (EDA)) *OR* (**title**: (vocalizat*)) *OR* (**title**: (gestur*)) *OR* (**title**: (acoustic*)) *OR* (**title**: (dermal*)) *OR* (**title**: ("autonomic nervous system")) *OR* (**title**: ("physiological arousal")) *OR* (**title**: (biomarker*))) *OR* ((**abstract**: ("language style")) *OR* (**abstract**: (facial)) *OR* (**abstract**: ("skin conductance")) *OR* (**abstract**: ("skin temperature*")) *OR* (**abstract**: (respirati*)) *OR* (**abstract**: ("heart rate")) *OR* (**abstract**: ("speech rate")) *OR* (**abstract**: (vocal)) *OR* (**abstract**: (pitch)) *OR* (**abstract**: (nonverbal)) *OR* (**abstract**: ("nonverbal")) *OR* (**abstract**: ("body movement")) *OR* (**abstract**: ("body language*")) *OR* (**abstract**: (EDA)) *OR* (**abstract**: (vocalizat*)) *OR* (**abstract**: (gestur*)) *OR* (**abstract**: (acoustic*)) *OR* (**abstract**: (dermal*)) *OR* (**abstract**: ("autonomic nervous system")) *OR* (**abstract**: ("physiological arousal")) *OR* (**abstract**: (biomarker*))) *OR* ((**KEYWORDS**: ("language style")) *OR* (**KEYWORDS**: (facial)) *OR* (**KEYWORDS**: ("skin conductance")) *OR* (**KEYWORDS**: ("skin temperature*")) *OR* (**KEYWORDS**: (respirati*)) *OR* (**KEYWORDS**: ("heart rate")) *OR* (**KEYWORDS**: ("speech rate")) *OR* (**KEYWORDS**: (vocal)) *OR* (**KEYWORDS**: (pitch)) *OR* (**KEYWORDS**: (nonverbal)) *OR* (**KEYWORDS**: ("nonverbal")) *OR* (**KEYWORDS**: ("body movement")) *OR* (**KEYWORDS**: ("body language*")) *OR* (**KEYWORDS**: (EDA)) *OR* (**KEYWORDS**: (vocalizat*)) *OR* (**KEYWORDS**: (gestur*)) *OR* (**KEYWORDS**: (acoustic*)) *OR* (**KEYWORDS**: (dermal*)) *OR* (**KEYWORDS**: ("autonomic nervous system")) *OR* (**KEYWORDS**: ("physiological arousal")) *OR* (**KEYWORDS**: (biomarker*))))) *AND* ((((**IndexTermsFilt**: ("Therapeutic Alliance")))) *OR* (((**title**: (relation*)) *OR* (**title**: (alliance*)) *OR* (**title**: (interaction*)) *OR* (**title**: (interpersonal*))) *OR* ((**abstract**: (relation*)) *OR* (**abstract**: (alliance*)) *OR* (**abstract**: (interaction*)) *OR* (**abstract**: (interpersonal*))) *OR* ((**KEYWORDS**: (relation*)) *OR* (**KEYWORDS**: (alliance*)) *OR* (**KEYWORDS**: (interaction*)) *OR* (**KEYWORDS**: (interpersonal*)))) *OR* (((**IndexTermsFilt**: ("Psychotherapeutic Processes")) *OR* (**IndexTermsFilt**: ("Countertransference")) *OR* (**IndexTermsFilt**: ("Psychotherapeutic Transference")) *OR* (**IndexTermsFilt**: ("Therapeutic Processes"))))) *AND* **Peer-Reviewed Journals only** | 589 |

Scopus

| # | Query | Results |
| --- | --- | --- |
| 5 | (TITLE-ABS-KEY (relation* OR alliance* OR interaction* OR interpersonal*)) AND (TITLE-ABS-KEY (therapist* OR psychotherapist* OR psychologist* OR psychiatrist* OR analyst)) AND (TITLE-ABS-KEY (rupture* OR repair* OR tension* OR resolution* OR resolve* OR confrontat* OR negotiat* OR conflict* OR deterioration*)) AND ( TITLE-ABS-KEY (synchron* OR coordinat* OR covariation* OR coheren* OR linkage* OR contagion* OR attune* OR align* OR concordance* OR mirror* OR "language style" OR facial OR "skin conductance" OR "skin temperature*" OR respirati* OR "heart rate" OR "speech rate" OR vocal OR pitch OR nonverbal OR "nonverbal" OR "body movement" OR "body language*" OR eda OR vocalizat* OR gestur* OR acoustic* OR dermal* OR "autonomic nervous system" OR "physiological arousal" OR biomarker*)) | 764 |
| 4 | TITLE-ABS-KEY (synchron* OR coordinat* OR covariation* OR coheren* OR linkage* OR contagion* OR attune* OR align* OR concordance* OR mirror* OR "language style" OR facial OR "skin conductance" OR "skin temperature*" OR respirati* OR "heart rate" OR "speech rate" OR vocal OR pitch OR nonverbal OR "nonverbal" OR "body movement" OR "body language*" OR eda OR vocalizat* OR gestur* OR acoustic* OR dermal* OR "autonomic nervous system" OR "physiological arousal" OR biomarker*) | 6,186,480 |
| 3 | TITLE-ABS-KEY (rupture* OR repair* OR tension* OR resolution* OR resolve* OR confrontat* OR negotiat* OR conflict* OR deterioration*) | 4,686,762 |
| 2 | TITLE-ABS-KEY (therapist* OR psychotherapist* OR psychologist* OR psychiatrist* OR analyst) | 290,573 |
| 1 | TITLE-ABS-KEY (relation* OR alliance* OR interaction* OR interpersonal*) | 11,563,729 |
